# Gamma-glutamyltransferases: exploring the complexity of a multi-functional family of enzymes

**DOI:** 10.3389/fphar.2014.00221

**Published:** 2014-09-30

**Authors:** Maria Franzini, Alfonso Pompella, Alessandro Corti

**Affiliations:** ^1^Cardiology and Cardiovascular Medicine Division, Fondazione Toscana G. Monasterio – CNRPisa, Italy; ^2^Institute of Life Sciences, Scuola Superiore Sant'AnnaPisa, Italy; ^3^Department of Translational Research and New Technologies in Medicine and Surgery, Medical School, University of PisaPisa, Italy

**Keywords:** gamma-glutamyltransferase, glutathione, gamma-glutamyltransferase structure, gamma-glutamyltransferase fractions, microvesicles, biotechnological applications, prodrugs

Gamma-glutamyltransferase (γ-GT; EC 2.3.2.2) is the sole enzyme activity capable of cleaving the gamma-glutamyl bond present in the structure of glutathione (GSH), the well known antioxidant tripeptide, and is present on the outer aspect of plasma membranes of virtually all mammalian cells. γ-GT–mediated cleavage is the first step in the degradation of extracellular GSH, which proceeds by the action of dipeptidases and makes thus precursor aminoacids (cysteine in the first place) available for reuptake and re-utilization by the cell for GSH resynthesis. On the other hand, γ-GT is also responsible for the processing of other substrates including GSH in their structure, such as major inflammatory mediators leukotriene C4 (LTC4) and S-nitroso-glutathione (GSNO), as well as adducts of GSH with xenobiotics and metabolites formed by the action of glutathione-S-transferases.

Due to its critical role in cellular GSH resupply, γ-GT has long been considered as just a part of cellular antioxidant defenses. However, prooxidant effects of γ-GT were also documented (Dominici et al., 2005), and the significance of this enzyme activity in cellular physiology and medicine has considerably expanded as its central role in modulation of overall redox equilibria of the cell has become increasingly clear. In their book *“Gamma-Glutamyl Transpeptidases: Structure and Function”* (Castellano and Merlino, 2013) the authors provide a comprehensive coverage of the state-of-art knowledge on many relevant aspects of the matter. After a brief general introduction, Chapter 2 provides an updated overview of the complex γ-GT gene structure, as well as of expression, induction and distribution of the enzyme in mammalian tissues. The following chapters are concerned with structure-function correlates, and start with an interesting section on the enzyme “autoprocessing,” i.e., the process by which precursor γ-GT protein—translated as a single polypeptide—exerts a proteolytic cleavage on its own structure thus yielding a large and a small subunits which then assemble together to give the mature enzyme dimer. To date structural characterization of human γ-GT1 (hGT1) has been hampered by its heavily glycosylated structure, as well as by its association with cell membranes, and most of the knowledge regarding enzyme structure indeed comes from investigations in bacteria. The text analyzes in detail the characteristics of enzyme active site and factors affecting substrate binding affinity, the reaction mechanism and its steps, the mechanisms of enzyme inhibition by several distinct compounds, the assays available to measure enzyme activity, the biochemical characteristics of the enzyme protein, etc. This part—Chapters 3 and 4—form the core of the book, in which the authors directly capitalize the competences developed in their own laboratory.

Nevertheless, the text is forcedly lacking an important aspect of the matter, i.e. the recently published characterization of macromolecular complexes carrying γ-GT in biological fluids. It has been in fact documented that γ-GT associates in plasma with macromolecular carriers having different molecular weights, densities and charges, and recently four distinct γ-GT fractions were identified (Franzini et al., 2013). A first characterization has shown that the “b-GGT” fraction consists of membrane microvesicles resembling exosomes (Fornaciari et al., 2014). Additional insights into hGT1 high-resolution crystal structure have also been recently reported (West et al., 2013), highlighting specific differences between bacterial and human architectures.

A better knowledge of γ-GT structure will undoubtly help to clarify its interactions with carriers and improve its clinical value and specificity as a disease biomarker. Chapter 5 (“γ-GT in Bioclinical”) indeed provides an overview of the pathophysiological roles played by γ-GT in human pathology. In clinical medicine, γ-GT plasma levels are considered a well established and sensitive index of hepato-biliary disease and alcohol abuse, but during the last decade the involvement of γ-GT in other important diseases has also been documented. A series of large-scale, prospective studies have shown that plasma γ-GT values are independent predictors of cardiovascular mortality, stroke, metabolic syndrome and diabetes, which appears to confirm the involvement of γ-GT in mechanisms of flogosis and cellular damage. Expression of γ-GT during cancer progression is a factor in drug resistance of cancer cells, e.g., to cisplatin. This part however is a less interesting section of the book, as the same topics were previously discussed in detail by others (Pompella et al., 2007; Corti et al., 2010).

The final Chapter 6 includes an accurate and compelling overview to the biotechnological and biomedical applications of γ-GT, exploiting the unique capability of this enzyme to cleave the gamma-glutamyl bond. For this reason, e.g., the addition of a gamma-glutamyl group to a drug molecule can make it inactive until the moment the residue is cleaved off by γ-GT. The examples described include the synthesis of gamma-D-glutamyl-L-tryptophan (a compound employed for treatment of tuberculosis) and phenylhydrazine derivatives, exhibiting antioxidant and immunostimulatory properties. The use of γ-GT from *Bacillus subtilis*—which can act as cephalosporin acylase—in the production of cephalosporins, and the possible use of γ-GT in the production of theanine are also discussed. The Authors might have as well cited the recent development of the GSH-containing arsenic trioxide–based pro-drug 4-(N-(S-glutathionylacetyl)amino)phenylarsinous acid (GSAO), which is selectively activated by γ-GT expressed by cancer cells and exerts a remarkable anti-angiogenic effect (Dilda et al., 2008).

In summary, the book by Castellano and Merlino covers a full spectrum of relevant topics regarding γ-GT biochemistry, pathophysiology and biotechnology. This concise text—integrated by a combination of comprehensive and useful tables, figures, and graphs reporting essential information in a clear and detailed way—can provide a precious introduction to γ-GT, a complex and rapidly developing field of interest for a wide readership of biochemists, biologists, and clinicians.

## Conflict of interest statement

The authors declare that the research was conducted in the absence of any commercial or financial relationships that could be construed as a potential conflict of interest.
